# Memory and Motor Coordination Improvement by Folic Acid Supplementation in Healthy Adult Male Rats

**Published:** 2012

**Authors:** Maryam Khombi Shooshtari, Ahmad Ali Moazedi, Gholam Ali Parham

**Affiliations:** 1 *Department of Biology, Faculty of Sciences, Shahid Chamran University, Ahvaz, Iran*; 2 *Department of Statistics, Faculty of Mathematic, Shahid Chamran University, Ahvaz, Iran*

**Keywords:** Folic acid, Memory, Motor coordination, Rat

## Abstract

**Objective(s):**

Previous studies have shown that vitamin B as well as folate supplementation has been implicated in cognitive and neurodegenerative disorders including Alzheimer’s and Parkinson's diseases. The aim of present study was to evaluate the effects of folic acid on passive avoidance task and motor coordination in healthy adult male rats.

**Materials and Methods:**

Animals were randomly divided into five groups with 10 in each. 1) Sham treated (Veh); received same volume of normal saline as folate vehicle, 2-5) Test groups; each received a single dose of folate (5, 10 and 15 mg/ml/kg, IP daily for one week). At the end of the treatment with folic acid or vehicle, motor coordination in rotarod (after 24 hr) and passive avoidance memory in shuttle box (after 2 and 30 days) were evaluated, respectively.

**Results:**

The results showed that folic acid (5, 10, and 15 mg/kg) increased short-term (*P*<0.05, *P*<0.001) memory while, long term memory affected significantly with doses 10 and 15 mg/kg (*P*<0.01, *P*<0.001). On the other hand, folic acid (5 and 10 mg/kg) had significant improving effect on motor coordination (*P*<0.001, *P*<0.01) but with 15 mg/kg dose didn't have any effect on motor coordination.

**Conclusion:**

Our results suggest that folic acid may improve both short- and long-term memories, dose dependently, although it affects motor balance at lower dose. The mechanism of folic acid effects on cognition and motor coordination is unknown and needs more investigations.

## Introduction

The relationships between diet, aging, and diseases are complex and provide a fertile ground for research that spans the disciplines of molecular and cellular biology, organ physiology, epidemiology, and clinical investigation (1). Folic acid (folate) is a water-soluble B-vitamin which exists in foods such as dried beans, peas, lentils, oranges, whole-wheat products, liver, asparagus, beet, broccoli, brussels sprouts and spinach. In some conditions such as cooking the fresh vegetables, their folate is degraded. For this reason, the losses should be compensated by nutritional supplementation (2). Folate participates in the transfer of 1-carbon unit (such as methyl, methylen, and formyl groups) to the essential substrates which involves in the synthesis of DNA, RNA and proteins (3, 4). Folic acid plays a role in the methylation of homocysteine providing the methyl group for the conversion of methionine to s-adenosyl methionine (3). An increase in homocysteine (Hcy) levels is a major consequence of folate deficiency that may have adverse effects on multiple organ systems during aging (1). Indeed, low folate intake increases plasma homocysteine, which damages the vascular endothelium and increases the risk of cardiovascular diseases. Homocysteine is also neurotoxic and hyperhomocysteinemia has been associated prospectively with higher risk of Alzheimer’s disease (5). Several lines of investigations showed that folate deficiency and elevated Hcy levels might also have adverse effects in adult nervous system and B vitamins, specifically folate, have been implicated in neurological disorders including those associated with cognitive disease (6, 7). Recently, several clinical and experimental studies have shown that there is an association between cognitive disorders and folic acid deficiency in normal aging as well as older subject (8-16). Conversely, high folate intake is associated with lower risk of Alzheimer's diseases (7, 17). *In vitro* studies on cell culture and also *in vivo* studies in animal models of neurodegenerative disorders have provided evidences that folate deficiency and elevated homocysteine levels could induce neuronal vulnerability, dysfunction and death (1). Previous studies have shown that vitamin B supplementation reduces homocysteine and enhances cognitive function in patients with mild cognitive disorders and low serum folic acid. However, whether folic acid enhances cognitive function in adult subjects without dementia and with normal serum folic acid is unknown (18). Folate deficiency in pregnant women who have experience abortion has led to an expanding research effort aimed at understanding the biological functions of folate in cellular metabolism and how folate deficiency adversely affects various types of cells during development (1, 19). The adverse effects of folate deficiency and elevated Hcy levels on brain development have been well documented (15). However, there is a little knowledge about the effects of folic acid on cognitive performance in normal adult subjects. Therefore, in this study, effects of folic acid on passive avoidance condition and motor coordination were investigated in healthy adult male rats. 

## Material and Methodsds

***Animals***

Forty adult male albino rats of Wistar strain (250±20 g, 3-4 months) obtained from animal centre laboratory of Ahvaz Jundishapur University of Medical Sciences (AJUMS) were used in this study. Animals were housed in standard cages under controlled room temperature (20±2°C), humidity (55-60%) and light exposure conditions 12:12 hr light–dark cycle (lighted on 07:00 a.m.). All experiments were carried out during the light phase of the cycle (8:00 a.m. to 6:00 p.m.). Access to food and water were *ad libitum* except during the experiments. Animal handling and experimental procedures were performed under observance of the University and Institutional legislation, controlled by the Local Ethics Committee for the Purpose of Control and Supervision of Experiments on Laboratory Animals. All efforts were made to minimize animal suffering and the number of animals used. Prior to the onset of behavioral testing, all rats were gently handled for 5 days (5 min daily).

Animals were randomly divided into five groups, consisting of 10 animals in each: 1) Four treated groups received intraperitoneal injections of each dose of folic acid once daily (0, 5, 10, and 15 mg/ml/kg) dissolved in normal saline for one week 2) sham treated group (Veh), which received the same volume of normal saline (1 ml as vehicle). Then, all groups were trained for passive avoidance learning and short- and long-term memories using two-way shuttle box apparatus. Twenty-four hr after training with inhibitory avoidance condition task, motor coordination of all rats were tested on rotarod. 

***Passive avoidance task***

The apparatus used for evaluation of the passive avoidance task was two-way shuttle box (Borj Sanaat Co. Iran), which consisted of two adjacent Plexiglas compartments of identical dimensions (27×14.5×14 cm). For the experimental procedure, on the first day (adaptation day) each rat was allowed a 3 min adaptation period and free access to either the light or dark compartment of the box to avoidance training and after being placed in a shuttle box. Following this adaptation period, on the second day (training phase), rats were placed in the illuminated compartment and 30 sec later the sliding door was raised. Upon entering the dark compartment, the door was closed and a 1.5 mA constant-current shock was applied for 3 sec. After 20 sec, the rat was removed from the dark compartment and placed into home cage. In order to test short- and long-term memories, 48 hr and 30 days after receiving foot shock, the rats were placed in illuminated chamber and 30 sec later the sliding door was raised and the latency of entering the dark compartment (step-through latency) and the time spent there during 5 min were recorded again, because the maximum time that was considered in this procedure was 300 sec (20-23).

***Rotarod test***

The rotarod test was performed in order to evaluate the motor coordination 24 hr after cognition tests in both treated and sham groups. Accelerating rotarod measures fine motor coordination, balanceand resistance to fatigue by assessing the duration that a rat can remain standing/walking on a rotating, slowly accelerating rod. After familiarizing rats with the instrument, the rotarod rotating speed was 5 rpm at the first 5 min and accelerated up to 40 rpm during the sec 5 min and remained constant after wards. Each rat was tested 3 times at day with 45 min intervals (24-26). Time spent (sec) on the rotarod was calculated for each rat (27).

***Statistics***

Data are expressed as mean±SEM of values for memory and motor coordination tests. Statistical analysis was performed by one-way ANOVA followed by LSD post-hoc test. A *P-value* less than 0.05 were assumed to denote a significant difference and levels of significance is indicated by symbols: *P<0.05, **P<0.01, ***P<0.001.

## Results

**Figure 1:**
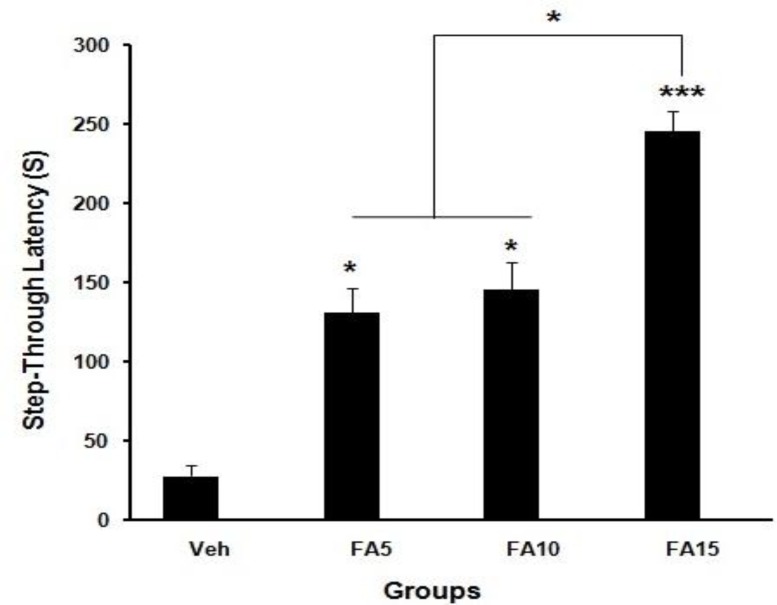
Effect of different doses of folic acid on step-through latency 48 hr after training (*P<0.05, ***P<0.001, n=10), (Veh: Sham treated, FA5: Folic acid 5 mg/kg/day, FA10: Folic acid 10 mg/kg/day, FA15: Folic acid 15 mg/kg/day)

**Figure 2:**
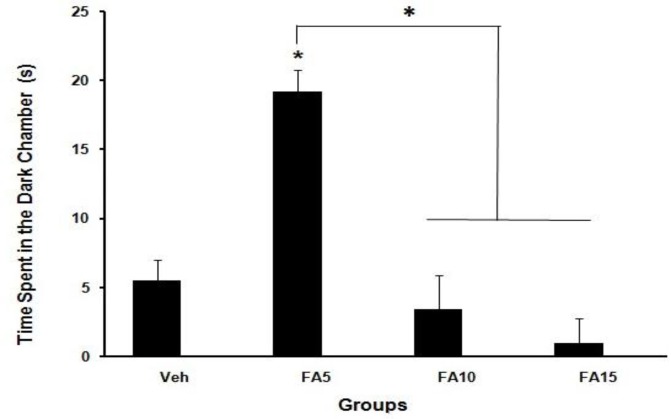
Effect of different doses of folic acid on step-through latency 30 days (long-term memory) after training (*P<0.05, **P<0.01, ***P<0.001, n=10), (Veh: Sham treated, FA5: Folic acid 5 mg/kg/day, FA10: Folic acid 10 mg/kg/day, FA15: Folic acid 15 mg/kg/day)

**Figure 3:**
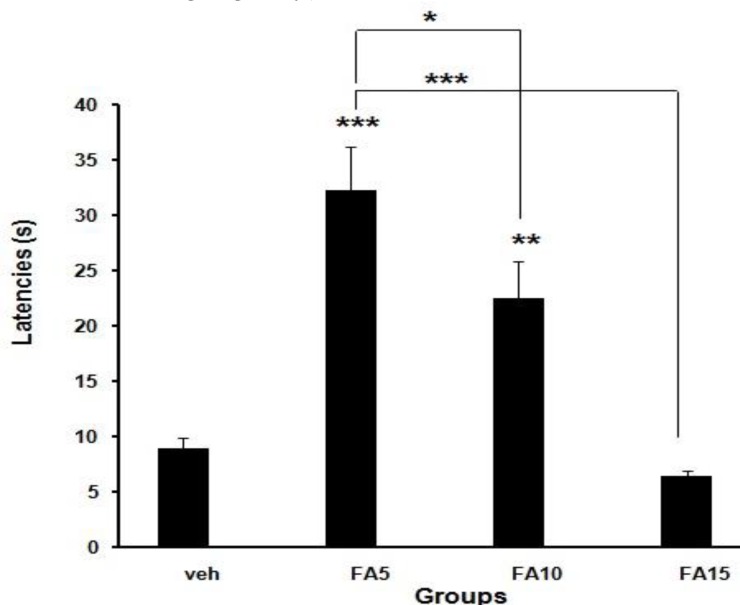
Effect of different doses of folic acid on time spent in the dark chamber 48 hr after training, (*P<0.05, n=10), (Veh: Sham treated, FA5: Folic acid 5 mg/kg/day, FA10: Folic acid 10 mg/kg/day, FA15: Folic acid 15 mg/kg/day)

**Figure 4:**
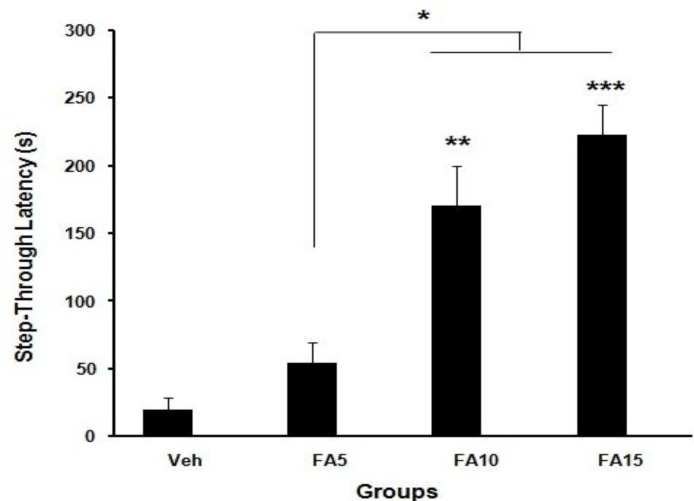
Effect of different doses of folic acid on time spent in the dark chamber 30 days after training (*P<0.05, n=10), (Veh: Sham treated, FA5: Folic acid 5 mg/kg/day, FA10: Folic acid 10 mg/kg/day, FA15: Folic acid 15 mg/kg/day)

Analyzed data of step-through latency (STL) of groups which were received folic acid (5, 10, and 15 mg/kg/day) and normal saline before training in the shuttle box showed that short-term memory improved significantly (*P*<0.05, *P*<0.001) (Figure 1). Step-through latency 30 days after training of rats which received folic acid (10 and 15 mg/kg/day) showed improved long-term memory significantly (*P*<0.01, *P*<0.001) when compared with sham group (Figure 2). Statistical analysis of the time spent in dark chamber at 48 hr after training showed that it was higher in rats which received 5 mg/kg/day folic acid compared with the sham group significantly (*P*<0.05) (Figure 3). However, 30 days after training, it was significantly decreased in rats which received 15 mg/kg/day folic acid when compared with the sham group (*P*<0.05) (Figure 4).

**Figure 5:**
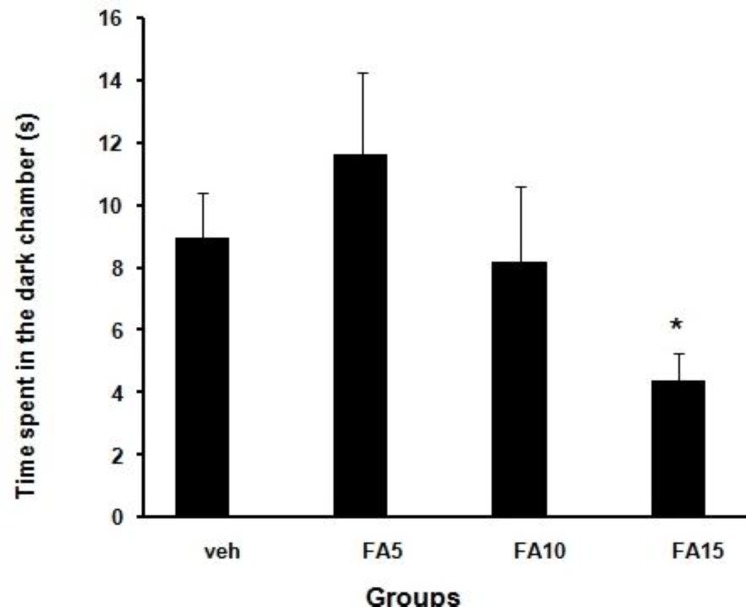
Means±SEM of bar descent latency following motor balance test in rotarod after different doses of folic acid supplementation or vehicle (*P<0.05, **P<0.01, ***P<0.001, n=10), (Veh: Sham treated, FA5: Folic acid 5 mg/kg/day, FA10: Folic acid 10 mg/kg/day, FA15: Folic acid 15 mg/kg/day)

Administration of 5 and 10 mg/kg/day folic acid increased motor coordination significantly (*P*<0.001, *P*<0.01), while 15 mg/kg/day folic acid had no significant effect on motor coordination in rotarod test (Figure 5).

## Discussion

The purpose of this study was to assess the potential effects of folic acid supplementation on passive avoidance memory and motor coordination in adult subjects. Our result showed that folic acid supplementation (5, 10, and 15 mg/kg/day) significantly improved short-term memory and also doses of 10 and 15 mg/kg/day significantly increased long-term memory. On the other hand, effect of folic acid (5 and 10 mg/kg/day, for one week) on motor coordination showed a significant improve on motor coordination. Therefore, the main finding of this study is that folic acid supplementation improved short-term and long-term memories and motor coordination in adult male rats in a dose-dependent manner. Folate has fundamental roles in central nervous system (CNS) functions (28) and is an important factor for normal development and function of the CNS in all ages (29). A study showed that the effect of folic acid supplementation is not restricted to the embryonic period but can also enhance growth, repair, and recovery in the injured CNS of adult rats (30). Evidence is increasing for beneficial and independent effects of folate on cognitive function (31). Folate deficiency in adults may increase the risk of coronary artery disease, stroke, several types of cancers and possibly Alzheimer's and Parkinson's disease (1). However, how folate deficiency impairs cognition is unknown (14). A randomized, double blind, placebo controlled study showed that daily supplementation of 800 μg of oral folic acid for 3 years increased serum folate concentrations, reduced total homocysteine level in plasma, and improved cognitive function. This finding suggests a close association between folic acid and homocysteine and cognitive function (32). Elevated homocysteine may increase risk of Alzheimer’s disease through its deleterious role in endothelial vascular pathogenesis as well as its direct neurotoxic effects. It potentiates the neurotoxicity of β-amyloid, enhances glutamate excitotoxicity, overstimulates *N*-methyl-D-aspartate (NMDA) receptors, and causes calcium influx into the neurons (5). Furthermore, a high homocysteine concentration as well as folate deficiency may decrease glutathione peroxidase activity and reduce tissue concentrations of antioxidant vitamins, making neurons more vulnerable to oxidative stress (5). In some cross-sectional studies, it was suggested that low serum folate and elevated plasma homocysteine concentrations decline cognition (12, 33). A review by Mattson and Shea described how folic acid and homocysteine were implicated in several neurological diseases. They cited evidence that folic acid might be important for DNA repair in post mitotic neurons. Furthermore, they stated hat homocysteine may induce damage in DNA of mature neurons, contributing to their damage and death. Possible explanations include increased intracellular calcium. Homocysteine may potentiate glutamate toxicity as well. Any or all of these factors might trigger apoptosis (8, 16). Luchsinger *et al* (2007) demonstrated that higher folate intake may decrease the risk of Alzheimer's disease independent of other risk factors such as of vitamins B_6_ and B_12_(8). However these results require confirmation with clinical trials (8). It has been postulated that the effects of folate deficiency on brain function is mediated by homocysteine. However, some studies showed an association between low folate status and cognitive impairment, thus dementia type of Alzheimer's disease remains significant after adjusting for confounding factors, so that folate may affect brain function through mechanisms that not directly are related to hyperhomocysteinemia (13). Previous studies showed beneficial effects of folic acid on cognitive performance and a relationship between folic acid deficiency and cognitive impairment. Therefore, this is the first study to evaluate the effects of normal levels of folic acid on passive avoidance performance in adult age subjects. This did not induce dopaminergic neurons death in mice with adequate folate intake, but it caused a significant decrease in dopaminergic neurons count and induced profound motor dysfunctions when combined with a folate-deficient diet (19). Certain findings suggest that folate intake is unlikely to be a major determinant of cognition risk; however they do not exclude the possibility of a mild to modest association between hyperhomocysteinemia and the risk of Parkinson’s disease (5). Prospective studies are necessary for examining the association between the risk of Parkinson’s disease and plasma concentrations of homocysteine, folate, vitamin B_6_, or vitamin B_12_. Furthermore, interactions between folate status and genetic polymorphisms of methylenetetrahydrofolate reductase should also be considered as individuals with these polymorphisms are more likely to have hyperhomocysteinemia, particularly; when combined with low folate status (7, 34). Lalonde *et al* (2008) expressed that elevated homocysteine levels resulted from vitamin B deficiencies have been hypothesized to contribute to the functional decline (7). 

## Conclusions

In summary, our results suggest that folic acid may improve both short- and long-term memories, dose dependently, while it affects motor balance at lower doses. The mechanism of folic acid effects on cognition and motor coordination is unknown and needs more investigations. However, it seems that folic acid supplementation involves pathogenesis of cognitive dysfunction and folic acid therapy may be used for treatment of cognitive and motor disorders. These findings require confirmation with more experimental studies.
